# Antibacterial and Healing Effect of Chicha Gum Hydrogel (*Sterculia striata*) with Nerolidol

**DOI:** 10.3390/ijms24032210

**Published:** 2023-01-22

**Authors:** Idglan Sá de Lima, Maria Onaira Gonçalves Ferreira, Esmeralda Maria Lustosa Barros, Marcia dos Santos Rizzo, Jailson de Araújo Santos, Alessandra Braga Ribeiro, Josy Anteveli Osajima Furtini, Edson C. Silva-Filho, Leticia M. Estevinho

**Affiliations:** 1 Interdisciplinary Laboratory of Advanced Materials (LIMAV), Postgraduate Program in Materials Science and Engineering, Federal University of Piauí, Teresina 64049-550, PI, Brazil; 2 Department of Biophysics and Physiology, Federal University of Piauí, Teresina 64049-550, PI, Brazil; 3 Centro de Biotecnologia e Química Fina (CBQF)—Laboratório Associado, Escola Superior de Biotecnologia, Universidade Católica Portuguesa, Rua Diogo Botelho 1327, 4169-005 Porto, Portugal; 4 Mountain Research Center, CIMO, Polytechnic Institute of Bragança, Campus Santa Apolónia, 5300-253 Bragança, Portugal

**Keywords:** antimicrobial, polymer, toxicity

## Abstract

Chicha gum is a natural polymer obtained from the *Sterculia striata* plant. The hydroxyl groups of its structure have a chemical affinity to form hydrogels, which favors the association with biologically active molecules, such as nerolidol. This association improves the biological properties and allows the material to be used in drug delivery systems. Chicha gum hydrogels associated with nerolidol were produced at two concentrations: 0.01 and 0.02 g mL^−1^. Then, the hydrogels were characterized by thermogravimetry (TG), Fourier Transform Infrared spectroscopy (FTIR), and rheological analysis. The antibacterial activity was tested against *Staphylococcus aureus* and *Escherichia coli*. The cytotoxicity was evaluated against *Artemia salina*. Finally, an in vivo healing assay was carried out. The infrared characterization indicated that interactions were formed during the gel reticulation. This implies the presence of nerolidol in the regions at 3100–3550 cm^−1^. The rheological properties changed with an increasing concentration of nerolidol, which resulted in less viscous materials. An antibacterial 83.6% growth inhibition effect was observed using the hydrogel with 0.02 g mL^−1^ nerolidol. The in vivo healing assay showed the practical activity of the hydrogels in the wound treatment, as the materials promoted efficient re-epithelialization. Therefore, it was concluded that the chicha hydrogels have the potential to be used as wound-healing products.

## 1. Introduction

The chicha (*Sterculia striata*) is an uncultivated fruit tree that is very abundant in nature. It is also known as xixá of cerrado, arachá, chichazeiro of cerrado, and macaque chestnut. This plant occurs in the Cerrado biome and is widely distributed in Brazil, mainly in the states of the Northeast and Midwest regions of the country [1,2].

Chicha gum, obtained from the exudate of the plant *Sterculia striata*, is produced by the plant to protect it from damage caused by external agents that damage the plant tissue or cause injuries. In addition, the exudate has the function of hydrating and reducing contamination by microorganisms in the injuries suffered by the plants. Rich in natural organic compounds, such as secondary metabolites, it aims to protect the plant from abiotic and biotic factors. The metabolites have nutritional and pharmacological values that provide the exudate with antimicrobial, germicidal, and protection properties against ultraviolet rays [3,4].

Chicha gum is chemically and functionally similar to karaya gum, which is widely used in the pharmaceutical industry as an emulsifier and binder. However, the karaya gum differs in the absence of xylose. Chicha gum, in its native form, has 10.7% of the acetyl group. When deacetylated, it presents 57.8% of neutral sugars, 23.4% of galactose, 28.8% of rhamnose, 5.6% of xylose, and 42.2% of uronic acid residues [4]. Chicha gum is a biopolymer with essential properties that allow for its use in different forms and compositions, such as gels and nanoparticles, for wound treatment, the prevention of skin infections, sunscreen, and drug release, in addition to adding value to the regional raw material [5].

The gums have been used to develop hydrogels due to their numerous desirable characteristics. Hydrogels have applicability in medicine, the cosmetics industry, agriculture, and biotechnology. This flexibility of using hydrogels for different purposes is due to the possibility of modifying their shape and volume, which depends on the applied stimulus, such as changes in pH and temperature. Furthermore, hydrogels are also responsive to magnetic and electrical fields [6,7,8,9]. In addition, these materials are hydrophilic, biocompatible, permeable, and show a low interfacial tension [10].

The similarity of chicha gum and karaya gum [1] makes this material attractive for biomedical areas. Furthermore, recent works indicate that the ethanolic extract of its stem bark has anti-inflammatory and contraceptive activity; thus, chicha gum can be associated with several biologically active molecules, enhancing its properties and value [11,12].

Essential oils, or extracts thereof, have promising biomedical applications and are among the biologically active molecules that can be associated with chicha gum. These oils have properties that are interesting for biomedical, chemical, and pharmaceutical applications. However, due to their unstable properties, they are generally not used in isolation. This limits some of their biological activities, requiring the association with materials capable of preserving or potentiating their microbial and antioxidant activities [13,14,15].

Nerolidol is an essential oil extract from citrus plants, such as kiwi and pepper [16]. It is an acyclic sesquiterpene that acts as an antineoplastic [17], in the defense against predators [18] as a healing agent [5], and as a potential enhancer of skin penetration for the transdermal administration of drugs [19], and has antimicrobial, leishmanicidal, larvicidal, and antioxidant activity [20,21].

Among the biological activities of essential oils, antimicrobial activity is one of the most important, considering that multidrug-resistant pathogenic bacteria are a worldwide health problem [22,23]. Thus, the search for new substances with antimicrobial properties that may help to treat bacterial infections has become urgent [24]. Furthermore, because of the potential of chicha hydrogel and nerolidol and considering the difficulty in finding natural products that can fight bacteria, these materials are viable alternatives for developing different products for healthcare applications, such as antimicrobial agents and healing.

Thus, this study aims to synthesize chicha hydrogels associated with 0.01 and 0.02 g mL^−1^ of nerolidol and investigate the antimicrobial properties and toxicity of the materials produced. In addition, we intend to study their chemical and rheological characteristics and in vivo healing activity, aiming at future applications for wound treatment.

## 2. Results and Discussion

### 2.1. Thermal Analysis

**Figure 1:**
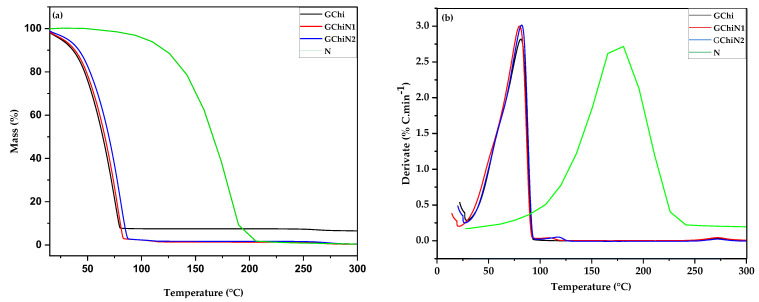
(**a**) TGA curves and (**b**) DTG curves of chicha hydrogel (GChi), nerolidol (N), chicha hydrogel with 0.01 g mL^−1^ nerolidol (GChiN1), and chicha hydrogel with 0.02 g mL^−1^ of nerolidol (GChiN2).

The thermogravimetric (TGA) and derivative (DTG) curves for pure chicha hydrogel and those associated with nerolidol (0.01 e 0.02 g mL^−1^) are illustrated in Figure 1. It is observed that nerolidol has a higher thermal stability compared to pure and associated hydrogels. Above 100 °C, nerolidol maintains approximately 100% of its mass, initiating a more significant loss of mass from 130 °C to 200 °C, with the peak of decomposition at 192.92 °C, when approximately 85% of its mass is degraded. Like nerolidol, chicha hydrogel (GChi) showed only one decomposition stage at a maximum decomposition temperature of approximately 80 °C. The decomposition of GChi happens up to 90 °C because the material has 98% water in its matrix (Figure 1a).

Figure 1b shows two thermal events for the chicha hydrogels associated with nerolidol (GChiN1, GChiN2). The first thermal event occurred in the range of 21.61 to 102 °C, a similar region shown for the pure chicha hydrogel. In addition, a second discrete event is observed in the temperature range from 102 to 128 °C for the associated hydrogels. This mass loss indicates the presence of nerolidol, which is more thermally stable than chicha. Since the GChiN2 sample has a higher amount of N than the GChiN1 sample, the second event occurred at a maximum decomposition temperature of 117.29 °C and, for GChiN1, at 107.98 °C, the series of mass being equal to 0.5%.

### 2.2. Spectroscopy in the Infrared Region

**Figure 2:**
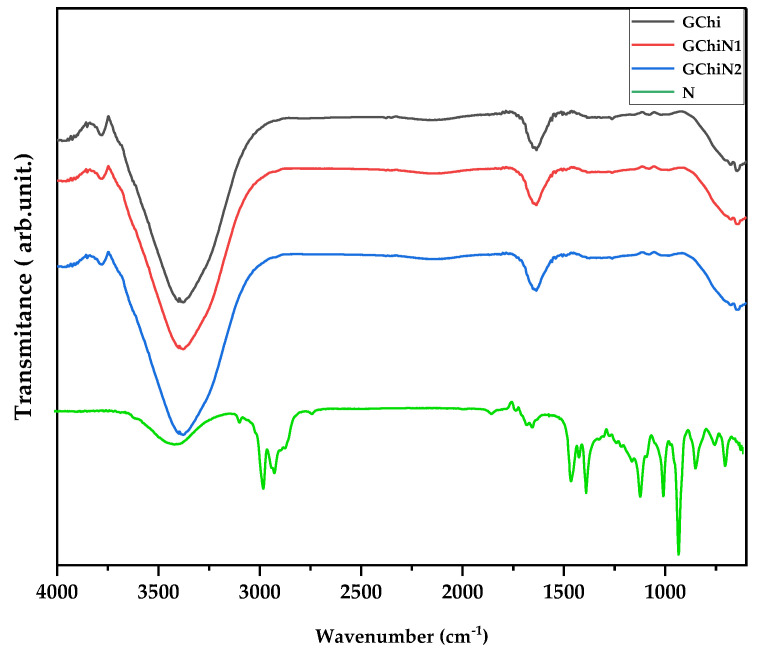
FTIR of chicha hydrogel (GChi), nerolidol (N), chicha hydrogel with 0.01 g mL^−1^ nerolidol (GChiN1), and chicha hydrogel with 0.02 g mL^−1^ nerolidol (GChiN2).

The FTIR spectra of chicha hydrogel (GChi), 0.01 g mL^−1^ nerolidol chicha hydrogel (GChiN1), 0.02 g mL^−1^ nerolidol Chicha hydrogel (GChiN2), and nerolidol (N) are shown in Figure 2.

The regions at 1750 cm^−1^, 1630 cm^−1^, and 1425 cm^−1^ are characterized by the presence of carboxylic groups, which is characteristic of gums from the genus Sterculia due to the presence of uronic acids [25]. In addition, for the chicha hydrogel, it is possible to visualize, in the region between 3550–3100 cm^−1^, the presence of axial deformation bands of N—H and the OH stretching vibration of the hydroxyls [25,26].

The profile of the derived hydrogels remains similar to the pure chicha hydrogel. However, an increase in intensity is observed in the regions previously described in the hydrogels GChiN1 and GChiN2 compared to GChi and pure nerolidol, which are related to the OH stretching vibrations in both materials. This result indicates the different interactions in the gel’s formation, with the presence of nerolidol in the bands referring to the region around 3100–3550 cm^−1^ [27].

The associated hydrogels did not show a band in the region of 2900 cm^−1^, which is characteristic of the methyl groups in nerolidol. The overlap of these bands can explain this result due to the enlargement in the region of 3500 cm^−1^ attributed to the hydroxyl groups present in the materials, both pure and associated. The region of 1500–1700 cm^−1^ is attributed to the deformation of the alkene groups in nerolidol. The region around 1400 cm^−1^ is attributed to an overlap of the alkane bands of the CH_2_ and CH_3_ groups in the N [5,28].

The FTIR results prove the interactions present in the materials. For example, it is possible to observe distinct regions of GChi and pure nerolidol in the materials formed by GChiN1 and GChiN2.

### 2.3. Mechanical Properties

The physical stability of a formulation and its properties, such as cohesion and adhesiveness, are essential parameters to be considered in the manufacture, storage, and application of topical products. Each product category must, therefore, present a rheological behavior suitable for the application [29,30]. Cohesion is a rheological property that expresses the ability of a material’s internal structure to maintain a strongly interconnected resistance without suffering rupture [31,32]. On the other hand, adhesiveness is related to the adhesion capacity of the material; in other words, the total force necessary for the sample to be separated from the probe [33]. Cohesion was calculated as the ratio of the positive area during compression and rupture in the hydrogel. Adhesiveness was calculated as the ratio of the negative area after hydrogel rupture and adhesion.

**Figure 3:**
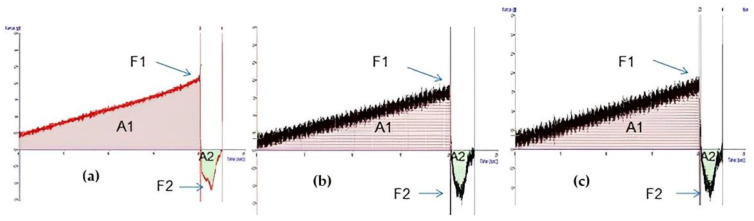
Texture analysis of hydrogels. A1 (Area 1), A2 (Area 2), F1 (Force 1), F2 (Force 2) of (**a**) chicha hydrogel (GChi), (**b**) chicha hydrogel with 0.01 g mL^−1^ nerolidol (GChiN1), and (**c**) chicha hydrogel with 0.02 g mL^−1^ nerolidol (GChiN2).

In a comparative analysis between the hydrogels GChi, GChiN1, and GChiN2, the highest values of cohesions and adhesiveness were observed in GChi samples, as shown in Table 1 and Figure 3. This result can be attributed to the rigidity of networks built by double cross-linking in the hydrogel’s matrix when nerolidol was introduced. These structures became weak, and the hydrogels were easily broken when tension was applied. In addition, the increased concentration of nerolidol implied a decrease in the hydrogel’s viscosity, cohesion, and adhesiveness. These results can be attributed to the presence of nerolidol in the synthesized hydrogels, which TG previously evidenced. The decrease in viscosity, cohesion, and adhesiveness may be beneficial in applying the hydrogel to wound care, as it may facilitate application to the injured area. Additionally, it may allow for a better interaction of the oil with the damaged tissue, allowing for a more effective drug distribution at the application site and improving the absorption and its therapeutic activity [34,35].

### 2.4. Toxicity to Artemia salina

The method using *Artemia salina* was proposed by Meyer [36] as a simple bioassay to determine the median lethal dose (LD50) of the substances tested. Artemia salina nauplii are used as a biological indicator of toxicity since they are non-selective mandatory filter feeders. These organisms filter out any residue, including those dissolved in the water. In addition, toxicity to this crustacean has been shown to correlate well with cytotoxic activity against human tumors [37].

LD50 values below 100 µg mL^−1^ indicate the product as being highly toxic, between 100 to 500 µg/mL indicates moderate toxicity, between 500 to 1000 µg mL^−1^ indicates low toxicity, and above 1000 µg/mL represents a non-toxic sample [32]. The toxicity test of pure chicha hydrogels ratified the results found by Braz [26]. The pure chicha hydrogel has an LD50 > 1000 μg mL^−1^. Therefore, it does not present toxicity. On the other hand, when associated with nerolidol, the hydrogels showed moderate toxicity, showing an LD50 of 100 μg/mL.

In their toxicity studies [20], using another model of acute experimentation in mice, the authors observed that nerolidol has a high LD50 value and is considered moderately toxic when administered in high doses. Another toxicity study [34] against *A. salina* with the species Montrichardia linifera demonstrated that the dichloromethane fraction showed a high toxicity against *A. salina* (LD50 < 31 μg mL^−1^) with antiplasmodic activity. These results indicate that the material can be promising in antimalarial activity. In addition, plant extracts and derivatives that have a high toxicity against *A. salina* demonstrate high potential for biological activities, being very useful for using this bioassay in phytochemical studies searching for bioactive substances.

The use of nerolidol associated with polymeric matrices shows promise. In studies carried out by [38], nerolidol associated with chitosan hydrogel was shown to be effective in the healing of skin wounds in mice without significant toxicity.

### 2.5. Antibacterial Activity

#### Direct Contact Test with the Pure Hydrogel and Associated with Nerolidol

**Figure 4:**
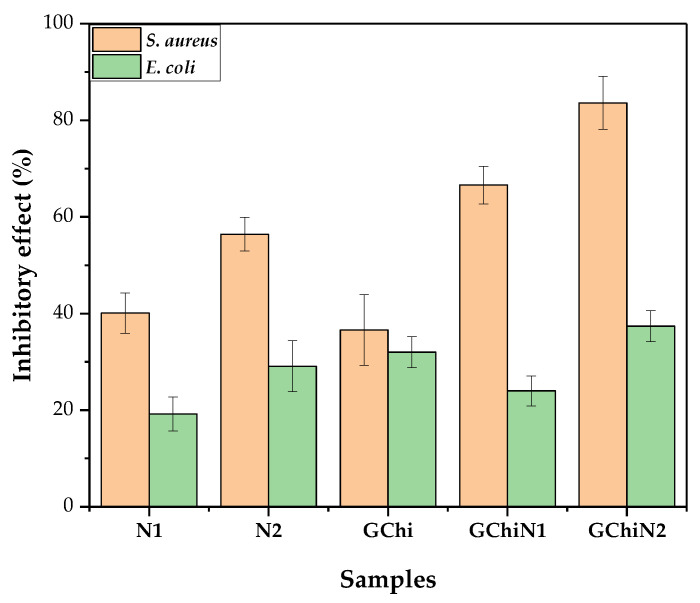
Antibacterial activity of GChi, N1, N2, GChiN1, and GChiN2 against *Staphylococcus aureus* (ATCC 25,923) and *Escherichia coli* (ATCC 10536).

**Figure 5:**
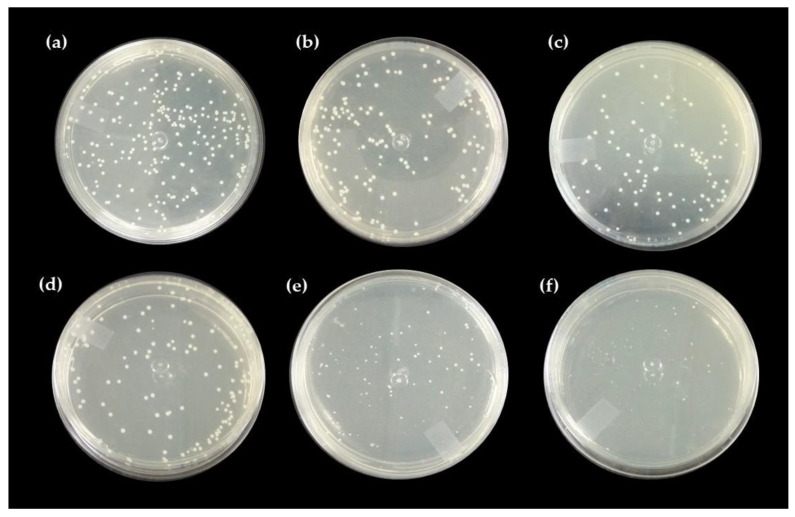
Direct contact test for (**a**) control, (**b**) N1, (**c**) N2, (**d**) GChi, (**e**) GChiN1, and (**f**) GChiN2 against *Staphylococcus aureus* (ATCC 25,923).

**Figure 6:**
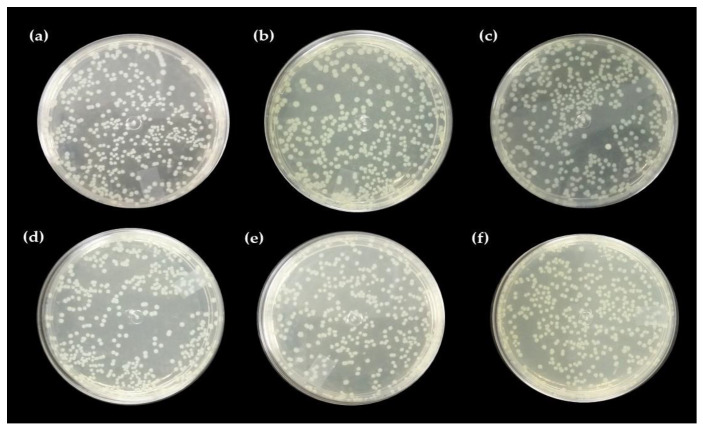
Direct contact test for (**a**) control, (**b**) N1, (**c**) N2, (**d**) GChi, (**e**) GChiN1, and (**f**) GChiN2 against *Escherichia coli* (ATCC 25922).

Figure 4, Figure 5 and Figure 6 show the results of chicha hydrogel (GChi), nerolidol 0.01 g mL^−1^ (N1), nerolidol 0.02 g mL^−1^ (N2), chicha hydrogel with N1, and chicha hydrogel with N2 against *S. aureus* and *E. coli* bacteria.

The associated hydrogels showed better results against the Gram-positive strain than pure chicha hydrogel and pure nerolidol in the different concentrations tested. Figure 5 shows that the antibacterial activity against the *S. aureus* strain of the associate materials increased concerning the hydrogel of chicha and nerolidol in the different concentrations (Figure 5).

Against the *E. coli* strain, the GChi, N1, and N2 materials showed a lower inhibitory efficiency than against the Gram-positive strain. Even so, they demonstrated similar behavior regarding the potentiation of the antibacterial action, with an increase in the inhibitory effect of the associated materials compared to the pure materials (Figure 6).

The incorporation of nerolidol in GChi, proven by the results of TG and DTG, provided better antibacterial results, showing a synergism between the properties of pure materials.

Studies on the antimicrobial action mechanism of natural gums show, that after contact of the gum with the microorganism, there is an increase in roughness and a decrease in cell size, which causes cell lysis and the release of intracellular content [26,39,40]. The antimicrobial activity of nerolidol is related to its hydrophobic character, as the hydrocarbon tails and alcohol groups can alter the functions of the bacterial cell membrane in addition to enhancing the permeation of drugs in the transdermal form [41].

Gram-negative bacteria are less sensitive to hydrophobic substances such as nerolidol than Gram-positive bacteria. In addition, the cell wall of Gram-negative bacteria is rich in polysaccharides, inhibiting the penetration of antimicrobial substances [15]. As they do not have an additional permeability barrier in the cell wall, the efficiency in inhibiting colony growth in direct contact tests and antibacterial activity is higher against *S. aureus*.

Ferreira [38], when promoting the association of chitosan hydrogel with nerolidol, found a higher inhibitory effect of the associated materials compared to the pure materials against *Staphylococcus aureus*. These results corroborate the results presented in our study, as the chicha hydrogel showed antibacterial activity against strains of Gram-positive bacteria. Furthermore, after association with nerolidol, the effect was improved, probably because nerolidol is a hydrophobic substance, allowing for the gums’ better adhesion to the bacterial cell wall.

### 2.6. Healing Test

An adhesive bandage developed with a hydrogel has characteristics for providing a humid environment, allowing oxygen to permeate, and decreasing the temperature of the exposed surface. These characteristics lead to an adjustment in the temperature and reduce pain [42].

Wound healing occurs through hemostasis, inflammation, proliferation, and remodeling. A healing process is a dynamic event that involves a coordinated action of resident and migrating cellular cells within the extracellular matrix. It is mediated by cytokine released at the injury site [42,43]. Hemostasis is a process that includes steps tightly regulated by coagulation proteins and platelet activation to form a platelet plug at the site of damage (primary hemostasis), a meshwork of fibrin (secondary hemostasis), and clot stabilization and resorption to promote vascular repair [44].

Hydrogels based on polysaccharides, such as chicha gum, are attractive for use as healing agents, especially as they are biocompatible and antimicrobial. In addition, in plants that have suffered an injury, the exudate that gives rise to chicha gum acts as a healing and protective agent. Thus, the potential use of chicha gum as a hydrogel for a solution in a pure and simple form is investigated, as well as associated with 0.02 g mL^−1^ nerolidol.

### 2.7. Macroscopic Analysis of the Wound

**Figure 7:**
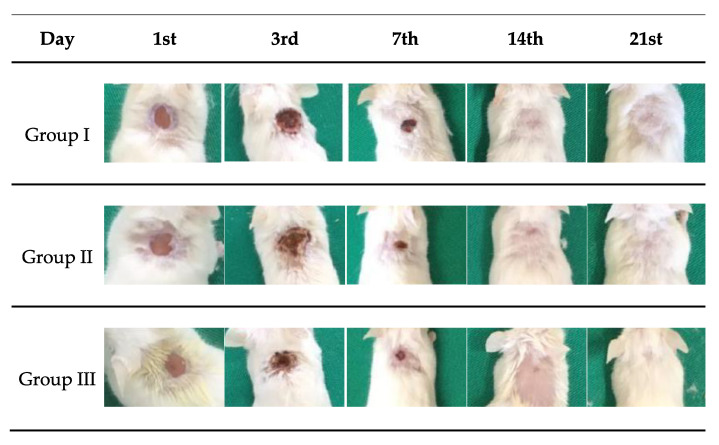
Macroscopic analysis of skin lesions in mice on 1st, 3rd, 7th, 14th, and 21st days of treatment with: Group I—physiological serum (PS); Group II—chicha hydrogel (GChi); and Group III—chicha hydrogel with 0.02 g mL^−1^ nerolidol (GChiN2).

Figure 7 shows the macroscopic images of skin wounds in mice treated with saline solution (PS), chicha hydrogel (GChi), and chicha hydrogel with 0.02 g mL^−1^ nerolidol (GChiN2). The concentration of nerolidol was chosen based on results reported in the literature, as described by Ferreira [38], where concentrations higher than 2% (*v*/*v*) can trigger inflammatory processes. In addition, the antibacterial activity in vitro previously reported that a concentration of 0.02 g mL^−1^ of nerolidol proved more efficient than chicha hydrogel with 0.01 g mL^−1^ nerolidol in Gram-positive and Gram-negative bacteria.

The macroscopic evolution of the wounds showed an excellent retraction of the lesion size. Normal hair growth was observed for groups I and II from the 14th day, and, for group III, only after the 21st day. On the 3rd day, serosanguineous exudate was observed in the animals from the three groups. For group III, which was treated with chicha hydrogel with 0.02 g mL^−1^ nerolidol, purulent exudate was observed in some animals’ wounds. This type of inflammatory exudate is indicative of wound contamination by pyogenic bacteria, which induces delays in the evolution of the healing process [45,46].

On the 7th day of evolution, it was possible to visualize the presence of a crust covering the wound’s surface in all animals from all groups. This crust helps to isolate the deeper portions of the skin from the outside environment and the proliferation of keratinocytes from each edge of the wound [47]. On the 14th day of treatment, animals in all experimental groups showed complete skin re-epithelialization.

**Figure 8:**
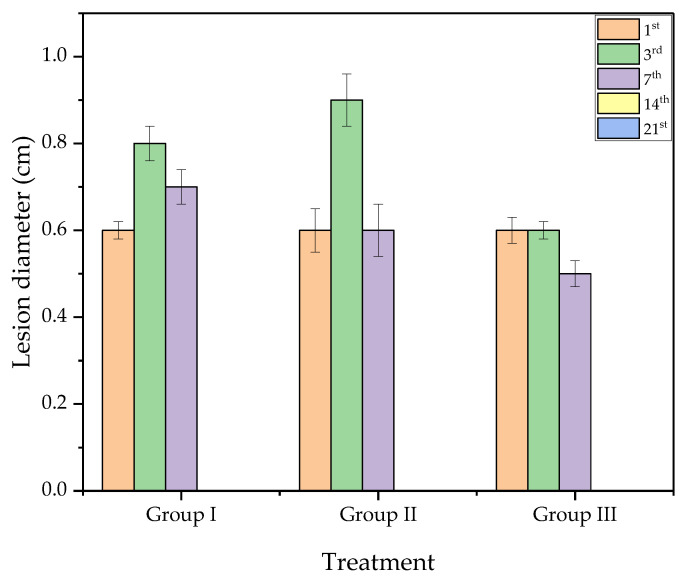
Evolution of the skin lesion diameter in mice on 1st, 3rd, 7th, 14th, and 21st days of treatment with: Group I—physiological serum (PS); Group II—chicha hydrogel (GChi); and Group III—chicha hydrogel with 0.02 g mL^−1^ nerolidol (GChiN2).

The evolution of the lesion diameter during the 21st day of treatment can be seen in Figure 8. On the third day, an increase in the size of the wound was observed in groups I and II. However, in group III (GChiN2), the diameter remained equal to 0.6 cm, as on the first day.

On the 7th day, the animals of all groups showed wound retraction. Animals from group III (GChiN2) showed a smaller diameter when compared to the first day, most likely due to the presence of myofibroblasts. From the 14th day, all wounds were closed entirely (Figure 7 and Figure 8). The complete re-epithelialization of the wound helps in the continuity of the healing process that still occurs in the deeper layers of the skin, preventing the entry of external microorganisms, which would delay the maturation of the granulation tissue due to the persistence of active inflammation at the wound site [48].

#### Qualitative Histological Analysis

**Figure 9:**
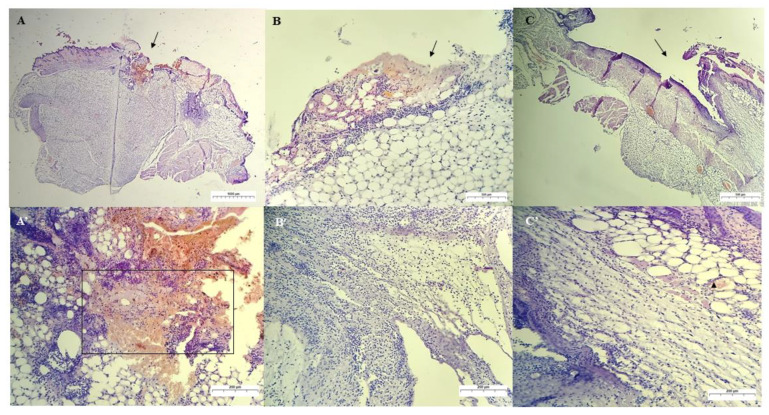
Photomicrograph of skin lesion in mice on the 3rd day of treatment. (**A**): Skin ulcer (arrow) treated with PS, showing in detail (**A’**) an area of cellular debris, clot, and intense PMN inflammatory infiltrate (magnification: 20× and 100×, respectively). (**B**): Skin ulcer treated with GChi, showing a smaller amount of fibrin clot (arrow), and, in detail (**B’**), an intense PMN inflammatory infiltrate through the layers of the deep dermis (magnification: 40× and 100×, respectively). (**C**): Cutaneous ulcer treated with GChiN2 (arrow), showing, in detail (**C’**), ulceration with cellular debris and mixed inflammatory infiltrate (PMN and MN) more concentrated around the wound, and with moderate infiltration in the dermis and blood vessel with leukocyte exudation (arrowhead—magnification: 40× and 100×, respectively). Staining by H.E. physiological serum (PS), chicha hydrogel (GChi), chicha hydrogel with 0.02 g mL^−1^ nerolidol (GChiN2), polymorphonuclear leukocytes (PMN), mononuclear leukocytes (MN).

Figure 9 shows all groups’ results for the 3rd day of treatment. In the animals of group I, which were treated with saline solution (control group), an intense inflammatory infiltrate was observed, which was predominantly polymorphonuclear. These infiltrates were observed around the wound’s edges and invading the deeper layers of the dermis and the fibrin clot. In addition, many degenerated neutrophils and cellular debris and a low number of macrophages were observed. These observations corroborate the findings of [49].

In group I, after three days of treatment, the wound was still not filled by granulation tissue since there were still much cellular debris, fibrin, degenerated neutrophils, and inflammatory cells migrating to the wound region.

In Figure 9, for the group treated with chicha hydrogel (GChi), the presence of hemorrhage and fibrin clot formation inside the wound was observed. This result was less intense than the control group. In addition, the presence of edema and aggregates of multifocal inflammatory cells, predominantly PMN (neutrophils), through the superficial dermis was noted, as well as a more profuse inflammatory infiltrate in the wound area that extends to the deep dermis. Bacterial colonies and a mild inflammatory process of the subcutaneous tissue were not observed.

Group III, treated with chicha hydrogel associated with 0.02 g mL^−1^ nerolidol (GChiN2), showed an intermediate concentration of inflammatory cells, with a milder mixed inflammatory infiltrate (PMN and MN) compared to the control group, but with a higher intensity of debris cells than group II. In addition, a clot and bacterial colony in the dermis indicate that GChiN2 could not prevent secondary wound infection. These results are consistent with the visualization of purulent exudate present in the skin lesion of the animals in this group. This finding is consistent with those found in significant skin defects, which are more predisposed to potential secondary infections and may delay the complete healing process of the wound [44,50]. During the removal of necrotic tissue by macrophages while performing phagocytosis, these cells release growth factors responsible for angiogenesis and fibroplasia for wound healing [47].

**Figure 10:**
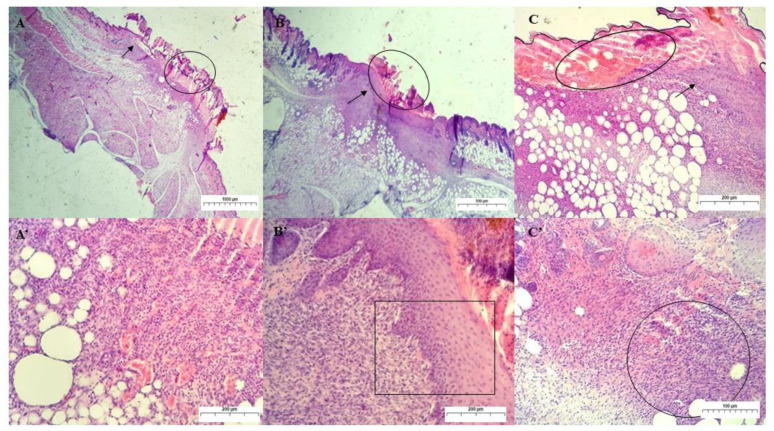
Photomicrograph of skin lesion in mice on the 7th day of treatment. (**A**): Granulation tissue (arrow) and crust (circle) in the wound treated with PS, showing in detail (**A’**) area of vascularized granulation tissue and intense MN inflammatory infiltrate (magnification: 20× and 100×, respectively). (**B**): Cutaneous wound treated with GChi showing the presence of acanthosis (arrow) and crust (circle), and in detail (**B’**), acanthosis (magnification: 40× and 100×, respectively). (**C**): Skin wound treated with GChiN2, where acanthosis is observed at the right edge of the border (arrow) and, in detail (**C’**), immature granulation tissue (magnification: 100× and 200×, respectively). Staining by H.E. physiological serum (PS), chicha hydrogel (GChi), chicha hydrogel with 0.02 g mL^−1^ nerolidol (GChiN2), polymorphonuclear leukocytes (PMN).

On the secondary union’s 7th day of treatment, the acanthosis process was observed in the animals from group II. The keratinocytes of the epidermis of each wound’s edges proliferate below the crust and unite in the midline, causing a thickening of the epidermal layer in the old lesion. This re-epithelialization event was completed in this group by the 7th day of treatment, whereas re-epithelialization in the control and III groups was still incomplete (Figure 10). During wound edge closure (epithelial regeneration), in the dermal layer, new vessels sprout (angiogenesis) that will for allow the growth of granulation tissue (fibroplasia) and subsequent maturation and remodeling of collagen fibers [51].

In the control and group III, no bacterial colonies were found during this treatment period. In addition, a decrease in the ulcer diameter was verified with the presence of the dehydrated clot (crust) on the surface of the lesion and the inflammatory infiltrate in the dermis region, characterized by the presence of monocytes [44,48].

In our study, we observed the presence of granulation tissue in the dermal layer in animals from all experimental groups on the 7th day. The immature granulation tissue showed high cellularity, with the proliferation of fibroblasts in the three groups, macrophages in large numbers, and clusters of neutrophils. Numerous newly formed vessels in this scar tissue were more intensely observed in group I, which had passive congestion and foci of hemorrhage.

**Figure 11:**
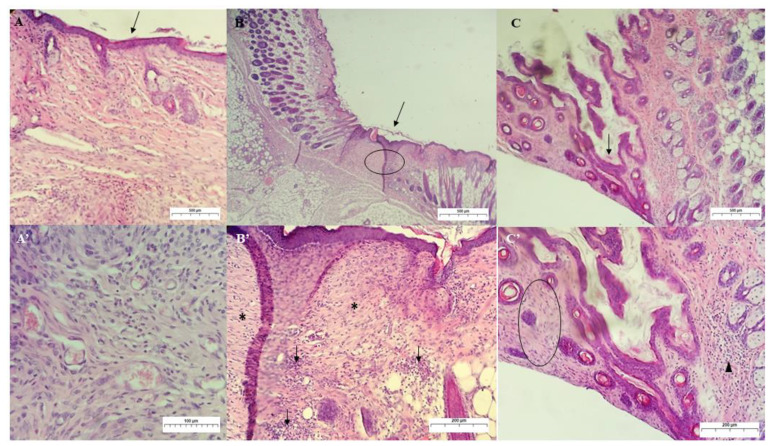
Photomicrograph of skin lesion in mice on the 14th day of treatment. (**A**): Re-epithelization (arrow) of the wound treated with PS, showing in detail (**A’**) granulation tissue in the deep dermis with several newly formed vessels (magnification: 40× and 200×, respectively). (**B**) Re-epithelization (arrow) and collagenization (circle) in the dermis for the GChi-treated group, showing in detail (**B’**) fibroblast proliferation (asterisks) and mixed inflammatory infiltrate (arrows) (magnification: 40× and 100×, respectively). (**C**): Re-epithelization (arrow) of the wound treated with GChiN2, in detail (**C’**), collagenization and fibroblasts (circle), and presence of hair follicles and mononuclear infiltrate (arrowhead) (magnification: 40× and 100×, respectively). Staining by H.E. physiological serum (PS), chicha hydrogel (GChi), chicha hydrogel with 0.02 g mL^−1^ nerolidol (GChiN2).

Figure 11 shows histopathological analyses of the control and experimental groups on the 14th day of treatment. For group I, the analysis revealed the presence of a thin layer of epidermis re-epithelialized in the region of the old wound and an extensive area of collagenization at the dermo-epidermal junction (non-individualized collagen fibers), with an infiltration of mononuclear cells and fibroblasts and a decrease in the number of newly formed vessels [47,52].

In group II, which showed a complete re-epithelialization of the epidermis on the 7th day of treatment, a thinner epidermal layer was observed on the 14th day, with the presence of more mature granulation tissue and with a more significant deposition of collagen fibers. The spatial arrangement of collagen fibers in the wound is shown mainly in the horizontal position. In addition, many fibroblasts and mononuclear inflammatory cells and some eosinophils are observed, and a few newly formed vessels are persistent.

On the 14th day of treatment, group III showed a completely re-epithelialized thin epidermal layer, with the dermal layer showing predominantly mononuclear inflammatory infiltrate and the presence of keratin-filled hair follicles (follicular keratosis) and follicular atrophy, which was reflected macroscopically in the skin with alopecia at the wound. Collagen fibers were observed in both horizontal and vertical positions. Compared to group II, the tissue is more immature due to a bacterial colony. Since bacterial contamination delays the healing process, the GChiN2 group could not prevent secondary contamination for a significant experimentally induced skin defect.

**Figure 12:**
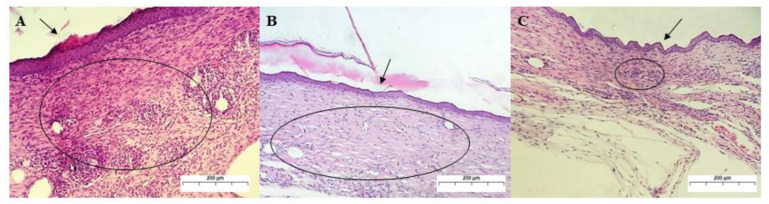
Photomicrograph of skin lesion in mice on the 21st day of treatment. (**A**): Re-epithelization and keratinization (arrow) of a wound treated with PS, with granulation tissue in the dermis (circle) still showing great cellularity and collagen deposition at the dermo-epidermal junction. (**B**): Thin layer of epidermal re-epithelization and keratinization (arrow) in the treatment with GChi, with granulation tissue in the mature dermis (circle) and few fibroblasts. (**C**): Re-epithelization and keratinization of the epidermis in the treatment with GChiN2, showing retraction of the wound, with avascular scar (circle) containing many spindle fibroblasts (magnification: 100×). Staining by H.E. physiological serum (PS), chicha hydrogel (GChi), chicha hydrogel with 0.02 g mL^−1^ nerolidol (GChiN2).

Figure 12 shows the analysis on the 21st day of treatment. Group I showed a complete re-epithelialization of the epidermis and a large collagen deposition area at the dermal–epidermal junction. In addition, a focal area of dystrophic calcification, moderate mononuclear inflammatory infiltrate, and fibroblast hyperplasia are observed, showing histological characteristics of a low wound contraction [53,54].

Group II showed a complete re-epithelialization of the epidermis, which had been observed since the 7th day of treatment. Mature granulation tissue was observed at the dermal–epidermal junction, and many mononuclear cells were in the mature granulation tissue. In group III, which also showed a complete re-epithelialization of the epidermis, focal agglomeration of PMN inflammatory infiltrates and the presence of MN cells at the dermo-epidermal interface were observed. The deposition of collagen fibers amid an extracellular matrix did not present linear formations and typical undulations, not allowing for their individualization [54,55].

## 3. Materials and Methods

### 3.1. Materials

The reagents used were: nerolidol (N), a mixture of cis- and trans-nerolidol geometric isomers, with 98% purity produced (SIGMA ALDRICH), magnesium chloride (MgCl) (IMPEX), calcium chloride (CaCl_2_) (DYNAMIC), sodium chloride (NaCl) (DYNAMIC), potassium chloride (KCl) (DYNAMIC), magnesium sulfate heptahydrate (MgSO_4_·7H_2_O) (VETEC), sodium bicarbonate (NaHCO_3_) (DYNAMICS), ethyl alcohol (C_2_H_5_OH) (ISOFAR), brain heart infusion (BHI) (HIMEDIA), Mueller–Hinton agar (HIMEDIA), resazurin (SIGMA), and dimethyl suffoxide (HIMEDIA) with high purity (98–99.9%). The water used in all processes and synthesis steps was distilled water. All reagents were used without prior purification.

### 3.2. Isolation and Purification of Chicha Gum

Chicha gum was isolated from nodules taken from chicha trees (Sterculia striata) at EMBRAPA-Meio Norte, in Teresina-PI, registered in the Herbarium Graziela Barroso, number TEPB:30418. As described by Rinaudo-Milas, chicha gum was isolated with some changes in the salt form [56]. First, the exudate (1.0 g) was dissolved in distilled water (100.0 mL) at room temperature for 24 h. To this solution, 1.0 g of NaCl was added and dissolved and filtered, and the pH was adjusted to 7.0 using 0.1 mol L^−1^ NaOH solution. Then, the polysaccharide precipitation was carried out with ethanol and washed with acetone. Finally, it was dried in an oven at 50 °C with hot air flow and maceration to obtain the powder, which was stored protected from light and humidity.

### 3.3. Synthesis of Hydrogels

Pure chicha hydrogel (GChi) was obtained from the reaction of chicha gum isolated with distilled water: 0.75 g of chicha gum for every 49.25 mL of distilled water. The solution was kept under magnetic rotation for 30 min.

#### Chicha Hydrogel with 0.01 e 0.02 g mL^−1^ Nerolidol

The chicha hydrogel associated with nerolidol 1 GChiN1) was synthesized by adding 0.5 mL of nerolidol (N) to 49.5 mL of GChi. Then, it was stirred for another 30 min. The same methodology was carried out to make 4.5 mL of nerolidol chicha hydrogel (GChiN2), with a proportion of 1 mL of nerolidol for every 4.5 mL.

### 3.4. Characterizations

A thermogravimetric study was carried out to investigate the thermal stability of the materials. The device used to obtain the results was the SDT Q600 V20.9 Build 20 device, model DSC-TGA Standard, with a heating rate of 10 °C min^−1^, a nitrogen atmosphere, and an alumina sample holder, varying the temperature from 0 to 300 °C, using approximately 10 mg. Infrared analyses (FTIR) were performed with the Varian 660-IR spectrophotometer in 1% KBr (m/m) of the sample in 32 scans in the region from 400 to 4000 cm^−1^ with a resolution of 4 cm^−1^.

The mechanical properties were determined in a TA-XT plus texture analyzer (Stable Micro Systems), operating in TPA (Texture Profile Analysis) mode. The samples were compressed twice by the cylindrical analytical test (10 mm), which penetrated to a depth of 10 mm in the sample, at a speed of 0.5 mm/s^−1^, with an interval of 5 s between compressions. With data from the curves of force versus time generated during the two compression cycles of the sample, it was possible to determine the hardness, adhesiveness, and cohesion values.

### 3.5. Cytoxicity Test against Artemia salina

#### 3.5.1. Synthetic Seawater Preparation

Synthetic seawater was prepared by adding 15.153 g of NaCl, 1.398 g of MgCl, 1.888 g of MgSO_4_, 0.652 g of CaCl_2_, 0.414 g of KCl, and 0.116 g of NaHCO_3_, and completed with distilled water until a final volume of 1.0 L [36].

#### 3.5.2. Toxicity Test

Following the methodology proposed by [36], in saline water at 12 ppm, the eggs of Artemia salina hatched, and the larvae were collected after 48 h to carry out the bioassays. Dilutions were performed with saline water and 0.5 mL of dimethyl sulfoxide.

All solutions were prepared in triplicate at concentrations of 1000, 100, 10, and 1 μg mL^−1^, and 10 nauplii of *A. salina* was added to the flasks. After 24 h, it was checked how many nauplii survived.

### 3.6. Antimicrobial Test

#### 3.6.1. Bacterial Lineage

The Gram-positive and Gram-negative bacterial strains, *Staphylococcus aureus* (ATCC 25923) and *Escherichia coli* ATCC 25922, respectively, used in the assays were provided by the Microbiology Research Laboratory of the Federal University of Piauí (UFPI).

#### 3.6.2. Inoculum Preparation

An aliquot of the bacterial growth was transferred to a falcon tube containing 3 mL of brain heart infusion (BHI) medium to obtain the bacterial cultures. The solution was incubated for 24 h at 37 °C. To obtain the bacterial inoculum, 1.0 mL of the culture and 9.0 mL of the BHI medium were mixed in a falcon tube.

#### 3.6.3. Antibacterial Activity by Direct Contact Method

Following the methodology described by [57], 100 μg mL^−1^ of the materials and 100 μL of the inoculum suspension standardized (1.5 × 10^8^ colony forming units per mL) were transferred to a Petri plate with Mueller–Hinton agar medium and seeded with the aid of a Drigalski. Plates were incubated for 24 h at 37 °C. In the positive control, only 100 μg/mL of inoculum was added. All assays were performed in triplicate.

The equation below was used to calculate the inhibitory effect:

(1) $$\eta =\frac{N_{1}-N_{2}}{N_{1}}\;\times 100\%$$

η = the inhibitory effect;

N_1_ = the arithmetic mean of the colony forming units of the control plates;

N_2_ = the arithmetic mean of the colony forming units of each of the tested solutions.

### 3.7. Healing Test

#### 3.7.1. Ethical Aspects

The research was carried out following the “Guide for the Care and Use of Laboratory Animals” recommendations (Institute of Laboratory Animals Resources, National Academy of Science, Washington, DC, USA, 2011). All procedures with animals obeyed ethical principles established by the National Council for the Control of Animal Experimentation Animals (CONCEA) and by the Ethics Committee in Animal Experimentation (CEUA) of UFPI following the provisions of Law 11,794 of 10.8.2008 and approved by the Ethics Committee of UFPI under protocol number 073/14.

#### 3.7.2. Animals

The animals used in this research were female mice of the Swiss lineage, aged 2–4 months and weighing between 25 and 30 g, obtained from the Central Animal House of the Agrarian Science Center—CCA of the Federal University of Piauí (UFPI). The animals were kept under a temperature equivalent to 26 ± 1 °C, with food and water ad libitum, and a 12 h light/dark cycle.

#### 3.7.3. Experimental Design

The animals were divided into three experimental groups, with 12 animals each: the animals treated with 0.9% saline solution (negative control) formed Group I; in Group II, treatment with chicha hydrogel (GChi) was carried out; and, in Group III, the animals were treated with chicha hydrogel associated with 0.02 g mL^−1^ nerolidol (GChiN2).

#### 3.7.4. Surgical Procedure

Before performing the skin wound, the mice were premedicated with anesthetics in a combination of 1/1 of xylazine hydrochloride (10 mg/kg) and ketamine hydrochloride (10 mg/kg) and administered intramuscularly. After anesthetic induction, each mouse was shaved and a circular piece of skin, 0.6 cm in diameter, was removed using a punch. At the end of the surgical procedure, the wound of each animal was treated daily, either with saline or hydrogels, and kept under daily observation.

#### 3.7.5. Wound Care

By topical route in the injured region, the animals were treated daily, administering hydrogels and saline solution in their respective groups [58].

#### 3.7.6. Euthanasia of Animals

Mice treated with 0.9% saline solution, with GChi and GChiN2 were euthanized at experimental times of 3 (*n* = 3), 7 (*n* = 3), 14 (*n* = 3), and 21 days (*n* = 3) post-surgical procedure to obtain the skin wounds. For the euthanasia procedure, the anesthetic association of xylazine (15 mg/kg: xylazine hydrochloride 2%) and ketamine (150 mg/kg: ketamine hydrochloride 50 mg/mL) was used and administered intraperitoneally (IP). Then, the excision of the skin fragment was performed for further histological analysis, ending the study with 21 days of treatment.

#### 3.7.7. Macroscopic Evaluation of the Skin Lesion

The animals were monitored daily for 21 days, observing macroscopic aspects such as wound healing, edema, exudate, and scab and wound color. In addition, the size of the lesions was verified with an analog caliper, and photographic records were taken on days 3, 7, 14, and 21 of the treatment.

#### 3.7.8. Qualitative Histological Evaluation of the Skin Lesion

A rectangular dorsum skin fragment measuring 0.5 × 1.0 cm was dissected from each animal after euthanasia. Each segment contained a central injured area and another peripheral area of intact skin. This procedure was repeated on the 3rd, 7th, 14th, and 21st days of treatment [59]. All samples of the skin lesions obtained were fixed in a 10% formaldehyde solution buffered with sodium hydroxide (pH around 7.0). They were kept for a maximum period of 48 h to subsequently undertake the usual histological routine, which constitutes dehydration, diaphanization, impregnation with paraffin, blockage, microtomy, and deparaffinization. Then, 4.0µm sections were made and adhered to glass slides, and then submitted to conventional histochemical staining (Hematoxylin-Eosin). After the procedure, the samples were analyzed by light microscopy (Opticam O500R).

The histological evaluation considered important parameters for tissue recovery, such as the presence of granulation tissue, inflammatory infiltrates, and re-epithelialization

## 4. Conclusions

The materials were obtained satisfactorily and efficiently. The characterization techniques proved the presence of nerolidol in the chicha hydrogels, as seen in the thermal degradation profiles. Texture analyses showed that the characteristics of the adhesion and cohesiveness of the hydrogels decreased as the concentration of nerolidol increased, making the material less cohesive and adhesive, and facilitating its application and the release of the active substance.

Chicha hydrogels associated with nerolidol showed significant action against the growth of the *S. aureus* strain, therefore being feasible for their use as an antimicrobial agent for topical use, even though they have shown a moderate toxic profile. In addition, the results of the healing test show improvements in the healing process, with GChiN2 showing better antimicrobial and healing action results. Thus, the synthesized material was easy and inexpensive to produce, making it economically attractive for biomedical applications.

## Figures and Tables

**Table 1:** Texture properties of chicha hydrogels (GChi) and chicha hydrogel with 0.01 g mL^−1^ nerolidol (GChiN1) and 0.02 g mL^−1^ nerolidol (GChiN2).

Sample	Area 1 [g s] (Cohesion)	Area 2 [g s] (Adhesiveness)	Force 1 [g] (Maximum Compression Force)	Force 2 [g] (Minimum Retraction Force)
GChi	23.3	−1.8	1.9	−2.9
GChiN1 N1	18.9	−1.6	1.9	−1.6
GChiN2	19.1	−1.4	1.9	−1.4

## Data Availability

The data presented in this study are available upon request to the author for correspondence.
